# Synthesis and Antimycobacterial Activity of 2,5-Disubstituted and 1,2,5-Trisubstituted Benzimidazoles

**DOI:** 10.3389/fchem.2020.00433

**Published:** 2020-06-19

**Authors:** Rogelio Jiménez-Juárez, Wendy Cruz-Chávez, Nayeli de Jesús-Ramírez, Guadalupe Ivonne Castro-Ramírez, Itzel Uribe-González, Gabriela Martínez-Mejía, Ricardo Ruiz-Nicolás, Charmina Aguirre-Alvarado, Nayeli Shantal Castrejón-Jiménez, Blanca Estela García-Pérez

**Affiliations:** ^1^Departamento de Química Orgánica, Escuela Nacional de Ciencias Biológicas, Instituto Politécnico Nacional, Mexico City, Mexico; ^2^Departamento de Microbiología, Escuela Nacional de Ciencias Biológicas, Instituto Politécnico Nacional, Mexico City, Mexico; ^3^Unidad de Investigación Médica en Inmunología e Infectología, Centro Médico Nacional, La Raza, IMSS, Mexico City, Mexico; ^4^Laboratorio de Bioquímica Farmacológica, Departamento de Bioquímica, Escuela Nacional de Ciencias Biológicas, Instituto Politécnico Nacional, Mexico City, Mexico; ^5^Área Académica de Medicina Veterinaria y Zootecnia, Instituto de Ciencias Agropecuarias-Universidad Autónoma del Estado de Hidalgo, Tulancingo, Mexico

**Keywords:** benzimidazole derivatives, *Mycobacterium tuberculosis*, mycobacterial intracellular activity, FtsZ protein, docking study

## Abstract

The appearance of drug-resistant strains of *Mycobacterium tuberculosis* and the dramatic increase in infection rates worldwide evidences the urgency of developing new and effective compounds for treating tuberculosis. Benzimidazoles represent one possible source of new compounds given that antimycobacterial activity has already been documented for some derivatives, such as those bearing electron-withdrawing groups. The aim of this study was to synthesize two series of benzimidazoles, di- and trisubstituted derivatives, and evaluate their antimycobacterial activity. Accordingly, **5a** and **5b** were synthesized from hydroxymoyl halides **3a** and **3b**, and nitro-substituted o-phenylenediamine **4**. Compound **11** was synthesized from an aromatic nitro compound, 4-chloro-1,2-phenylenediamine **9**, mixed with 3-nitrobenzaldehyde **10**, and bentonite clay. Although the synthesis of **11** has already been reported, its antimycobacterial activity is herein examined for the first time. 1,2,5-trisubstituted benzimidazoles **7a**, **7b**, and **12** were obtained from N-alkylation of **5a**, **5b**, and **11**. All benzimidazole derivatives were characterized by FT-IR, NMR, and HR-MS, and then screened for their *in vitro* antimycobacterial effect against the *M. tuberculosis* H37Rv strain. The N-alkylated molecules (**7a**, **7b**, and **12**) generated very limited *in vitro* inhibition of mycobacterial growth. The benzimidazoles (**5a**, **5b**, and **11**) showed *in vitro* potency against mycobacteria, reflected in minimal inhibitory concentration (MIC) values in the range of 6.25–25 μg/mL. Consequently, only the 2,5-disubstituted benzimidazoles were assessed for biological activity on mouse macrophages infected with *M. tuberculosis*. A good effect was found for the three compounds. The cytotoxicity assay revealed very low toxicity for all the test compounds against the macrophage cell line. According to the docking study, 2,5-disubstituted benzimidazoles exhibit high affinity for an interdomain cleft that plays a key role in the GTP-dependent polymerization of the filamentous temperature-sensitive Z (FtsZ) protein. The ability of different benzimidazoles to impede FtsZ polymerization is reportedly related to their antimycobacterial activity. On the other hand, the 1,2,5-trisubstituted benzimidazoles docked to the N-terminal of the protein, close to the GTP binding domain, and did not show strong binding energies. Overall, **5a**, **5b**, and **11** proved to be good candidates for *in vivo* testing to determine their potential for treating tuberculosis.

**Graphical Abstract:**
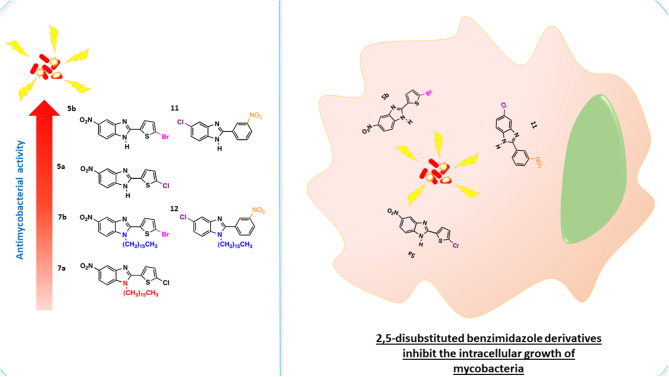


## Introduction

Tuberculosis, an infectious disease caused by the bacillus *Mycobacterium tuberculosis*, is the most widespread infectious disease worldwide. In 2018, 1.2 million deaths were attributed to tuberculosis and 10 million new cases emerged, according to the World Health Organization (WHO). Thus, it poses a serious threat to global health. *M. tuberculosis* is the deadliest infectious agent and one of the top 10 causes of mortality worldwide (World Health Organization (WHO), 2019). The appearance and spread of drug-resistant strains of this bacterium is an important reason for the reemergence of tuberculosis. The treatment of drug-resistant *M. tuberculosis* requires combinations of second- and third-line drugs. Apart from being less effective, these drugs are more expensive and more toxic. Hence, there is an urgent need to develop new compounds that can provide better results against susceptible and resistant tuberculosis strains.

The relevant strategies for developing new antimycobacterial drugs (Rode et al., 2019) are comprised of two approaches:

the chemical modification of a known molecule to enhance its biological activity (Thiagarajan et al., 2013; Sutherland et al., 2018), and the design and development of new molecules with antimycobacterial activity (Goverdhan et al., 2015; Lokesh et al., 2016). Within the framework of the former, the benzimidazole system is a scaffold for the development of new organic compounds with potential biological effects. Benzimidazoles have demonstrated antiulcer (Patil et al., 2008), anthelmintic (Dubey and Sanyal, 2010), antihypertensive (Naik et al., 2010), anticoagulant (Mederski et al., 2004), anti-inflammatory (Mader et al., 2008), antimicrobial (Lam et al., 2014), and antifungal (Alp et al., 2009) activity. The antimycobacterial effect of benzimidazole derivatives is also well-documented (Kazimierczuk et al., 2005; Stanley et al., 2012; Gong et al., 2014). An interesting review was published recently about promising antimycobacterial candidates formed by using a range of substituents at the 1-, 2-, 5-, and 6-positions of the benzimidazole core (Keri et al., 2016). An increase in the biological activity of benzimidazole has been achieved by making substitutions with electron-withdrawing groups (Klimesová et al., 2002; Pytela and Klimešová, 2011; Roh et al., 2017).

Although the mechanism of action of benzimidazole is not well understood, at least three plausible hypotheses have been made. Firstly, benzimidazoles are structural isosteres to purines (Arjmand et al., 2005) and thus may inhibit the biosynthesis of nucleic acids and proteins by competing with these heterocycles, leading to the death of bacterial cells (Zhang et al., 2013). Secondly, these derivatives can efficiently inhibit bacterial topoisomerases (Nimesh et al., 2014; Liu et al., 2018). Thirdly, the filamentous temperature-sensitive Z (FtsZ) protein, a tubulin homolog (van den Ent et al., 2001), has been explored as a molecular target for benzimidazoles, finding that the antimycobacterial activity of different benzimidazoles is related to their ability to impede FtsZ polymerization (Awasthi et al., 2013; Park et al., 2014).

The aim of the present study was to synthesize two series of 2,5-disubstituted benzimidazoles, including one previously reported compound, and test them for antimycobacterial activity. The six test compounds all bear electron-withdrawing groups. We hypothesized that an increase in the lipophilicity of the lead compound could enhance its biological activity since the *M. tuberculosis* cell wall is rich in lipids (Daffé, 2015). Accordingly, a linear aliphatic chain of 16 carbons was chemically linked to the N-1 position of the 2,5-disubstituted benzimidazoles to generate the series of 1,2,5-trisubstituted benzimidazoles. After being screened with an *in vitro* evaluation of their capacity to inhibit the *M. tuberculosis* H37Rv strain, the three best compounds were tested on a mouse macrophage cell line infected with *M. tuberculosis*. Because some of the benzimidazoles demonstrated antimicrobial activity against *M. tuberculosis*, the potential mechanism of action was explored by molecular docking simulations.

## Results and Discussion

### Chemistry

Benzimidazoles are recognized as an excellent platform for the development of new molecules with potential biological activity. Taking into account that the new molecules should have a simple, easily-prepared chemically structure, the test compounds were herein synthesized by environmentally friendly methods based on short and efficient synthesis procedures.

Among the reported methods for obtaining benzimidazoles, some utilize harsh (Gobis et al., 2015; Aghapoor et al., 2019) and others soft reaction conditions (Leutbecher et al., 2011; Sriramoju et al., 2018). Whereas, the former is carried out at a high temperature and with strong acids, the second is performed at or near room temperature (rt) in the presence of soft acids, mainly through heterogeneous catalysis. The first series of benzimidazoles herein synthesized was elaborated in two stages with a short, efficient, and sustainable method. The N-alkylation reaction used to form the second series required a longer synthetic procedure and led to low yields.

**Scheme 1:**
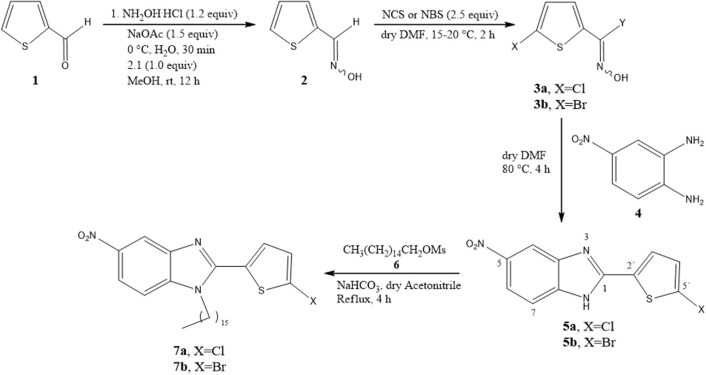
Synthesis of benzimidazoles **5a**/**5b** and **7a**/**7b** by using 2-thiophenhydroxymoyl halides **3a**/**3b** as key intermediates.

The procedure published by Abdelhamid et al. (1988) was modified to synthesize the new benzimidazoles, **5a** and **5b**. In order to substitute a nitro group at the 5-position of **3a** and **3b** (containing Cl and Br, respectively), the two 2-thiophenehydroxymoyl halides were treated with 4-nitro-1,2-phenylenediamine [**4**] in dry DMF at 80°C for 6 h (Scheme 1). Then the 2,5-disubstituted benzimidazoles were N-alkylated with hexadecyl methanesulfonate in basic medium at 120°C for 4 h to provide the 1,2,5-trisubstituted benzimidazoles (**7a** and **7b**) (Scheme 1).

2-Thiophenecarboxaldehyde oxime **2** was synthesized following the procedure described by Iwakura et al. (1968) to give the desired product as a white solid in 93% yield (mp 137–138°C, reported as 136°C) (Iwakura et al., 1968). In its FT-IR spectrum, oxime **2** showed a strong and broad band at 3,025 cm^−1^ attributed to the NO-H bond, and a band at 1632 due to the C=NOH double bond (Figure S1). In its ^1^H NMR spectrum, the compound exhibited two singlets at 11.68 and 7.68, corresponding to the hydroxyl and oxime protons, respectively, and three doublets of doublets at 7.54, 7.35, and 7.05 ppm assigned to H_5_, H_3_, and H_4_, respectively (Figure S2). In its ^13^C NMR spectrum, it displayed signals at 139.4, 131.0, 130.2, 130.1, and 125.6 ppm (Figure S3). The ^1^H NMR data were similar to those in the literature (Langa et al., 2000). On the other hand, 5-chlorothiophene-2-carbohydroximoyl chloride (**3a**) and 5-bromothiophene-2-carbohydroxamoyl bromide (**3b**) were synthesized with a modified protocol of Kanemasa (Kanemasa et al., 2000) by using 3 molar equivalents of N-chlorosuccinimide (NCS) or N-bromosuccinimide (NBS) relative to the oxime. Compound **3a** was afforded as a yellow solid in 95% yield (mp 129.5°C, similar to the 127–128°C reported by Iwakura et al.) (Iwakura et al., 1968), and **3b** was furnished as a brown oil in 93% yield.

One of the new benzimidazoles, **5a**, was obtained as an amorphous beige solid in 60% yield (mp 262–263°C). In the FT-IR spectrum of **5a**, there were strong bands at 3,253, 1,576, 1,504, and 1,338 cm^−1^, corresponding to the H-N, C=N, and NO_2_ groups, respectively (Figure S4). In its ^1^H NMR spectrum, the benzimidazole ring generated a doublet at (δ) 8.44 (*J* = 2.4 Hz for H_4_), a doublet of doublets at 8.17 (*J* = 8.7 and 2.4 Hz for H_6_), and a doublet at 7.72 (*J* = 9.7 Hz for H_7_), while the thiophene system produced two doublets at 7.76 and 7.19 (*J* = 3.9 Hz) for ${\text{H}}_{3^{\text{′}}}$ and ${\text{H}}_{4^{\text{′}}}$, respectively (Figure S5). In the ^13^C NMR spectrum, resonances for 11 aromatic carbons were observed at δ (ppm): 150.5, 144.5, 132.6, 128.9, 128.2, 119.7, 119.5, 118.8, 115.8, 112.1, and 108.5 (Figure S6). The LR-MS spectrum of benzimidazole **5a** revealed the expected pattern of molecular ions at 279 M and 281 M+2. The HR-MS spectrum allowed for the determination of an exact experimental mass of 279.9811 (M+H) (Figure S7) and an exact calculated mass of 279.9947 (M+H), thus confirming the structure of benzimidazole **5a** (Figure S8).

The other new benzimidazole, **5b**, was provided as an amorphous brown solid in 98% yield (mp 254–255°C). In the FT-IR spectrum of **5b**, the strong and broad band at [υ (cm^−1^)] 3,385 cm^−1^ corresponded to the N-H bond, and the strong bands at 1,625, 1,573, and 1,337 cm^−1^ to the C=N and NO_2_ groups (Figure S9). The ^1^H NMR spectrum showed a singlet [δ (ppm)] at 8.38 (for H_4_), a doublet at 8.09 (*J* = 7 Hz for H_6_), a singlet at 7.70 (for H_7_ and ${\text{H}}_{3^{\text{′}}}$), and another singlet at 7.38 (for ${\text{H}}_{4^{\text{′}}}$) (Figure S10). In the ^13^C NMR spectrum, only nine signals were found [δ (ppm)], at 150.1, 142.8, 133.9, 132.0, 128.9, 118.2, 116.1, 114.4, and 111.8] (Figure S11). In the LR-MS spectrum, **5b** displayed molecular ions at 323 and 325 due to the presence of a bromine atom (Figure S12). Based on the HR-MS spectrum, an exact experimental mass of 323.0410 was established, as was the exact calculated mass of 322.9364 (Figure S13).

The 1,2,5-trisubstituted benzimidazoles **7a** and **7b** were obtained by N-alkylation with the hexadecyl methanesulfonate in basic medium by heating at 124°C for 3 h, giving the regioisomer **7a** as a beige amorphous solid in 35% yield (704 mg) (mp 94–95°C). Its IR (KBr) spectrum exhibited the following absorption bands [υ(cm^−1^)]: two very strong bands at 2,916 and 2,848 corresponding to the aliphatic chain, a weak band at 1,520 owing to C=N, and bands at 1,472 and 1,462 attributed to the NO_2_ group. The ^1^H NMR spectrum showed the following signals at δ (ppm): a singlet signal at 8.31 for H_4_, a broad doublet of doublets at 8.21, the coupling constant (*J* = 10 Hz) for H_5_, and three doublets at 7.79 (*J* = 10 Hz), 7.41, and 7.05 (*J* = 5 Hz) assigned to H_7_, H_3_, and H_4_, respectively. Additionally, the triplet at 3.62 (*J* = 5.0 Hz) was ascribed to the methylene protons next to the benzimidazole nitrogen, a very broad singlet at 1.33–1.24 associated with protons of the aliphatic chain, and finally a triplet signal at 0.86 (*J* = 5 Hz) corresponding to protons of the methyl group (Figure S14).

The regioisomer **7b** was furnished as a beige amorphous solid in 30% yield (202.9 mg) (mp 93–94°C). In the IR (KBr) spectrum, the absorptions bands [υ (cm-1)] consisted of: two very strong bands at 2,917 and 2,850 due to C-H of the aliphatic chain, a weak band at 1,614 owing to C=N, and two bands at 1,470 and 1,331 caused by the NO_2_ group. The ^1^H NMR spectrum displayed the following signals at δ (ppm): a broad singlet at 8.39 attributed to H_4_, a broad triplet of doublets at 8.28 (*J* = 5 and 10 Hz) assigned to H_5_, a doublet at 7.88 (*J* = 10 Hz) corresponding to H_7_, two doublets at 7.73 and 7.69 (*J* = 5 Hz) ascribed to ${\text{H}}_{3^{\text{′}}}$ and ${\text{H}}_{4^{\text{′}}}$, a triplet at 3.98 (*J* = 5 Hz) owing to H_2_CN, a very broad singlet from 1.41–1.31 due to aliphatic chain protons, and finally, a triplet at 0.93 (*J* = 5 Hz) produced by methyl group protons (Figure S15).

**Scheme 2:**
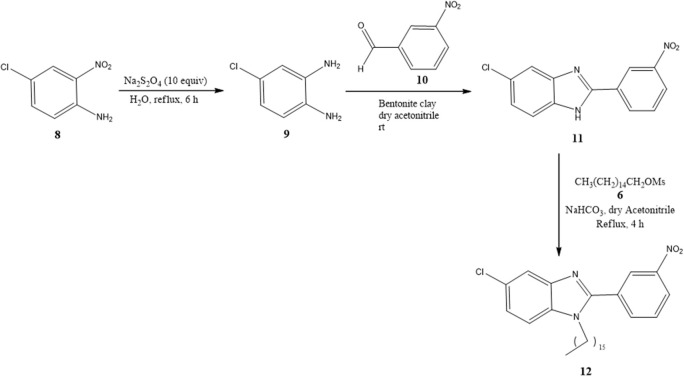
Synthesis of benzimidazoles **11** and **12** with bentonite clay serving as a heterogeneous catalyst.

Benzimidazoles **11** and **12** were synthesized by adopting recently published environmentally friendly protocols (Cardozo et al., 2015) with **9** as a key intermediate. Thus, nitro compound **8** was reduced with sodium metabisulfite in aqueous medium and heated to reflux to give 4-chloro-1,2-phenylenediamine **9**. Compound **9** was obtained as an amorphous beige solid (mp 173–174°C) (Park et al., 2014) in quantitative yield. The spectroscopic data were similar to results documented in the literature (Cantillo et al., 2013). 4-Chloro-1,2-phenylenediamine **9** (without purification) was reacted with 3-nitrobenzaldehyde **10** and bentonite clay, the latter as a heterogeneous catalyst, under constant stirring at rt (Scheme 2) (Mckinney et al., 1962; Naeimi and Alishahi, 2014). 2-(3′-Nitrophenyl)-5-chlorobenzimidazole (**11**) was produced as an amorphous beige solid in 64% yield (mp 243–244°C) as described by Keurulainen et al. (2010) (mp 243°C).

Its FT-IR spectrum showed strong bands at υ: 3,322, 1,513, and 1,347 cm^−1^, corresponding to the N-H and NO_2_ groups. In the ^1^H NMR spectrum of benzimidazole **11**, the phenyl group exhibited triplets (δ) at 8.96 (*J* = 1.2 Hz for ${\text{H}}_{2^{\text{′}}}$) and 7.82 (*J* = 7.8 Hz for ${\text{H}}_{5^{\text{′}}}$), and doublets of triplets at 8.55 (*J* = 7.8 and 1.2 Hz for ${\text{H}}_{6^{\text{′}}}$) and 8.31 (*J* = 7.8 and 1.2 Hz for ${\text{H}}_{4^{\text{′}}}$). On the other hand, the protons of the benzimidazole ring generated a triplet at (δ) 7.64 (*J* = 9.0 Hz for H_4_ and H_7_) and a doublet of doublets at 7.24 (*J* = 8.4 Hz for H_6_). In the ^13^C NMR spectrum, there were eight resonances for 13 carbons. The LR-MS spectrum of benzimidazole **11** displayed the expected pattern with molecular ions at 273 (M) and 275 (M+2). Spectroscopic and spectrometric properties were similar to those reported previously (Keurulainen et al., 2010).

The trisubstituted benzimidazole **12** was prepared by N-alkylation of **11** in hexadecyl methanesulfonate 6 basic medium and heating at 120°C for 4 h to provide an amorphous beige solid in 18% yield (36 mg) (mp 95–96°C). In the ^1^H NMR spectrum, the following signals can be appreciated at δ (ppm): a broad singlet at 8.59 assigned to ${\text{H}}_{2^{\text{′}}}$, a broad doublet of doublets at 8.39, (*J* = 5 Hz) attributed to ${\text{H}}_{4^{\text{′}}}$, two broad doublets at 8.13 (*J* = 5 Hz) and 7.81 (*J* = 5 Hz) ascribed to ${\text{H}}_{6^{\text{′}}}$ and H_4_, respectively, a triplet at 7.75 (*J* = 5 Hz) corresponding to ${\text{H}}_{5^{\text{′}}}$, a triplet at 7.36 (*J* = 10 Hz) and 7.34 (10 Hz) owing to ${\text{H}}_{5^{\text{′}}}$ and H_7_, a triplet at 3.64 (*J* = 5 Hz) associated with the methylene protons next to the benzimidazole nitrogen, a quintuplet at 1.57 (*J* = 5 Hz) due to β-methylene protons close to the benzimidazole nitrogen, a very broad singlet at 1.26 caused by protons of the aliphatic chain, and finally, a triplet at 0.88 (*J* = 5 Hz) generated by methyl group protons (Figure S16).

### Antimicrobial Activity

The benzimidazole scaffold is attractive for drug design because of the diverse activities of its derivatives. Benzimidazoles substituted with electron-withdrawing groups, such as nitro (Pytela and Klimešová, 2011; Roh et al., 2017) and halogen groups (Cl, F, and Br), have shown enhanced biological activity (Klimesová et al., 2002). The disubstituted and trisubstituted benzimidazole molecules herein synthesized were evaluated for their *in vitro* antimicrobial effect on *M. tuberculosis* H37Rv. The *in vitro* data revealed that the 2,5-disubstituted benzimidazoles were more efficient than the 1,2,5-trisubstituted compounds in their capacity to kill the mycobacterium. The benzimidazole derivatives capable of effectively inhibiting the growth of *M. tuberculosis* were **5a**, **5b**, and **11**, with MIC values of 89.6, 19.4, and 22.9 nM, respectively (Table 1). Contrarily, low antimycobacterial activity was detected for the molecules trisubstituted at the 1-position with the aliphatic chain, **7b**, **7a**, and **12**, with MIC values of >182, >198, and >201 nM, respectively.

**Table 1:** Antimycobacterial activity of 2,5-disubstituted and 1,2,5-trisubstituted benzimidazoles.

**Abbreviation**	**Benzimidazole derivatives**	**MIC μg/ml (nM)**
		**Antimycobacterial activity (H37Rv)**
**5a**	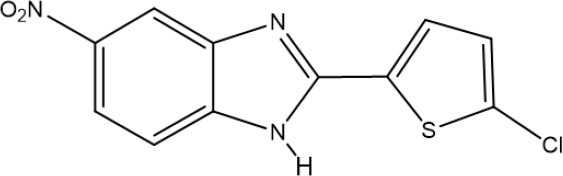	25 (89.6)
**7a**	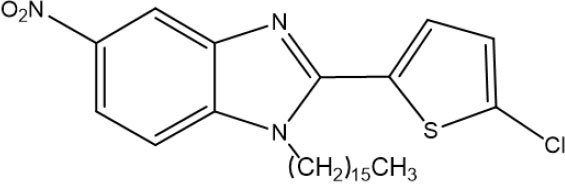	>100 (>198)
**5b**	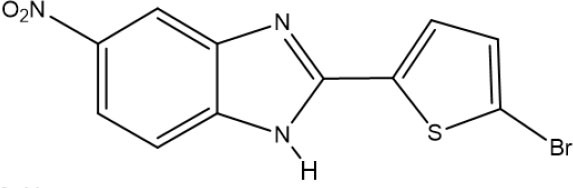	6.25 (19.4)
**7b**	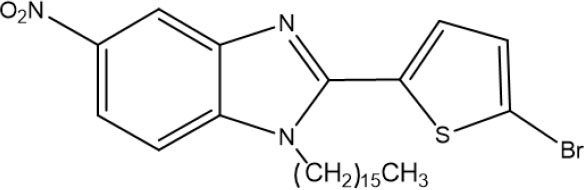	>50 (>182)
**11**	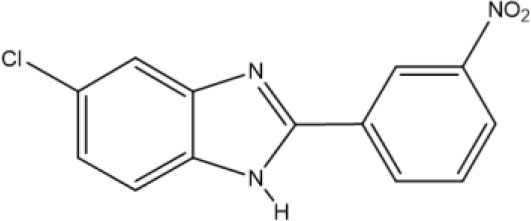	6.25 (22.9)
**12**	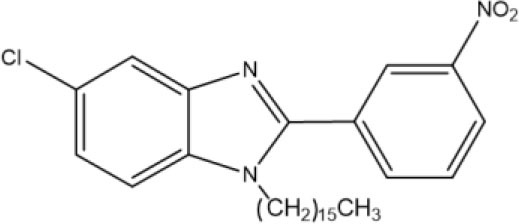	>50 (>201)

*Isoniazid (INH) = 0.125 μg/ml (0.9 nM)*.

**Figure 1:**
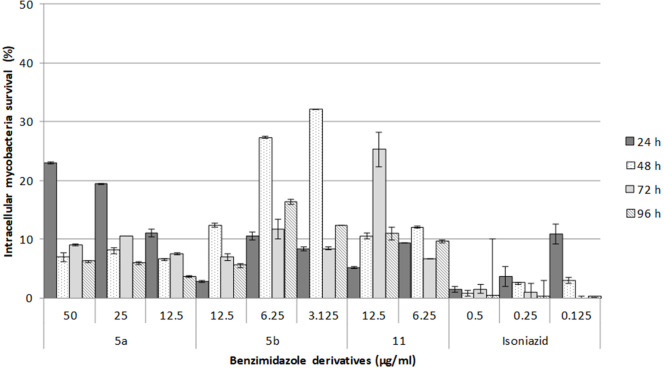
Survival of intracellular mycobacteria in macrophages treated with each of the three 2,5-disubstituted benzimidazole derivatives. In independent assays, macrophages were infected for 2 h and one of the benzimidazole derivatives or isoniazid was added. Intracellular growth was then monitored at 96 h post-infection. The data are expressed as the mean ± standard error of two measurements.

Based on the *in vitro* results, the toxicity of **5a**, **5b**, and **11** was tested on murine macrophages infected with *M. tuberculosis* (Figure 1). This intracellular bacillus mainly resides in alveolar macrophages and type II pneumocytes. Although only moderate antimycobacterial activity was found for **5a** *in vitro* (25 μg/mL MIC), it displayed a good intracellular effect. After 48 h of treatment, only 10% of mycobacteria were viable, demonstrating the inhibition of mycobacterial replication, similar to the type of activity reported for isoniazid. Compounds **5b** and **11** exhibited a moderate intracellular effect. After exposure to **5b** and **11**, only 30% of the mycobacteria could recover. Hence, these compounds also impeded bacterial replication, and their toxicity toward mycobacteria was lower than that of **5a**. The data published for **11** on the inhibition of the intracellular *C. pneumoniae* are similar to the current findings (Keurulainen et al., 2010).

### Cytotoxicity Assay

**Figure 2:**
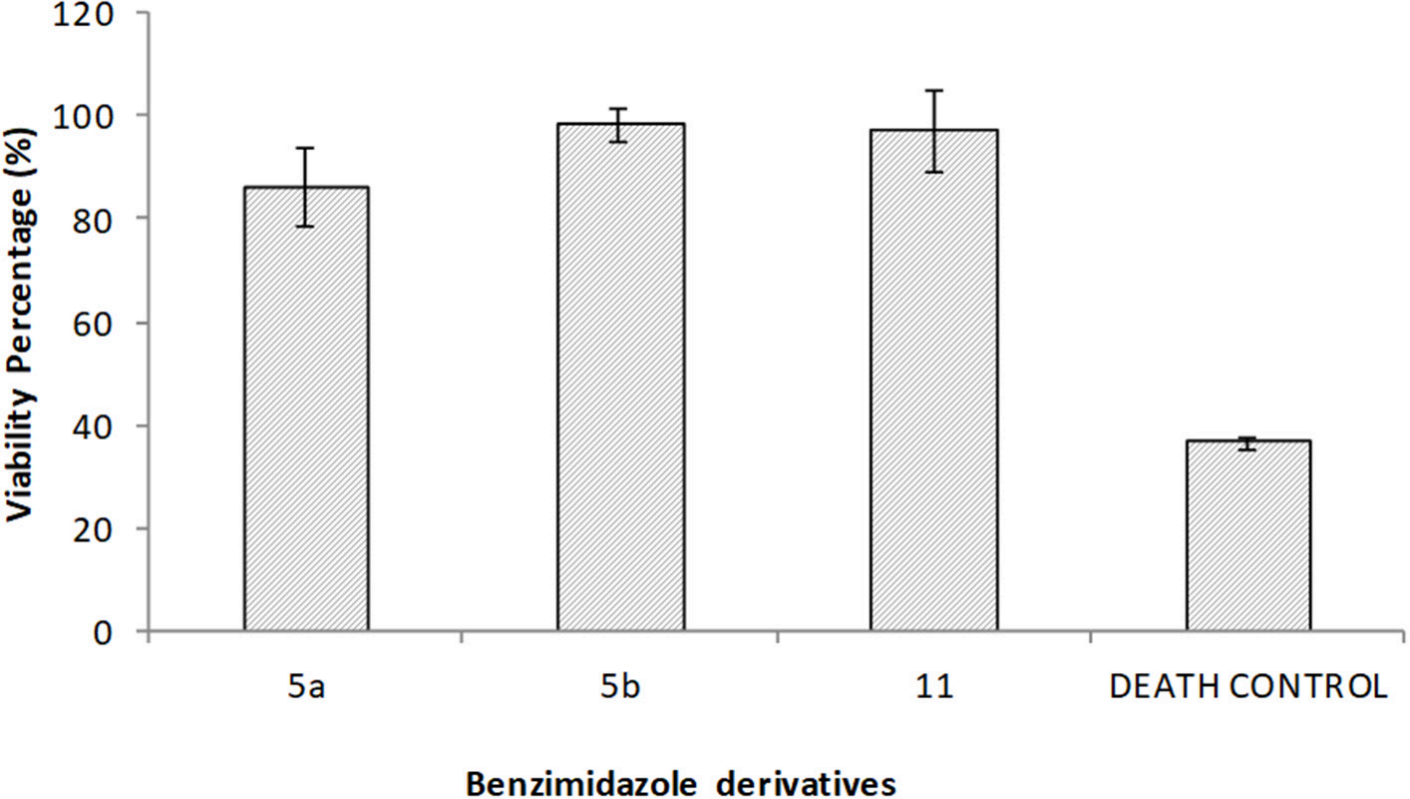
Cytotoxicity was evaluated for the 2,5-disubstituted benzimidazole derivatives on macrophages. In independent assays, macrophage monolayers were exposed during 24 h to 50 μg/ml of each one of the benzimidazole solutions, 40 μg/ml ursolic acid as the death control, or left untreated for the viability control. Values represent the mean ± standard deviation of two independent experiments. No significant differences were found between benzimidazole derivatives.

For the cytotoxicity assays, macrophages were exposed to the benzimidazole derivatives at concentrations higher than the MIC for 24 h. Subsequently, the viability of the cells was determined with Alamar Blue. None of the compounds showed any significant cytotoxic effect on macrophages (Figure 2). Even **5a**, with the lowest viability (86%), is not precluded from being used as an antimycobacterial agent.

### Structure Activity Relationship (SAR)

Since nitro and halogen groups are known to enhance the biological activity of pharmacophores (Salar et al., 2017), examination was presently made of the effect of different electron-withdrawing groups (2-haloheteroaryl or nitroaryl, 5-chloro or nitro) at positions two and five of the benzimidazole core. The disubstituted (**5a**, **5b**, and **11**) but not the trisubstituted (**7a**, **7b**, and **12**) benzimidazoles produced good antimycobacterial activity against *M. tuberculosis*. Compounds **5b** and **11** induced a better effect than **5a** (19.4 nM and 22.9 nM, respectively, versus 89.6 nM). The former two molecules each contain two electron-withdrawing groups, and a cooperative effect between them may explain the robust antimycobacterial activity. In relation to the two electron-withdrawing groups of **5b**, there is one bromo at the C-5′ position of the 2-(thien-2′-yl) and a nitro group at the C-5 position of the benzimidazole ring. On the other hand, **11** bears a nitro group at the C-3′ position of 2-(p-3'-nitrophenyl-1'-yl) and a chloro group at the C-5 position of the benzimidazole ring. Compound **5a**, an analog of **5b**, has a chloro group at the C-5'position of 2-(thien-2'-yl) instead of a bromo group, and a nitro group at the C-5 position of benzimidazole ring. Its lower antimycobacterial potency possibly owes itself to a less efficient cooperative effect of the substituents against *M. tuberculosis*. The charge of the cell wall of *M. tuberculosis* is negative (Hoque et al., 2012; Phillips et al., 2017), while that of the 2,5-disubstituted benzimidazoles is likely to be positive, due to the acceptor ability of these compounds for nitrogen atoms. Hence, another plausible hypothesis about the efficient antimycobacterial activity of the 2,5-disubstituted benzimidazoles is their potential ability to interact with the cell wall of mycobacteria through electrostatic charges.

In the case of 1,2,5-trisubstituted benzimidazoles, we hypothesized that the long aliphatic chain of sixteen carbon atoms bonded on the 1-position of the benzimidazole ring might improve the non-covalent intermolecular interaction with the highly lipophilic cell wall of the mycobacterium (Daffé, 2015), leading to an increase in antimycobacterial activity. According to the current data, however, the introduction of the aliphatic chain was detrimental to such activity. Similarly, the alkyl chains in novel aminopyrimidinyl benzimidazole were reported to reduce antibacterial potency (Liu et al., 2018).

The difference in the biological activity between the two series of benzimidazoles may stem from their distinct solubility properties. The 2,5-disubstituted benzimidazoles were soluble in dimethylsulfoxide and the 1,2,5-trisubstituted benzimidazoles in chloroform, owing to the strong influence in the latter compounds of a hydrophilic/hydrophobic imbalance, being predominant the hydrophobic effect. In this sense, a shorter aliphatic chain for the 1,2,5-trisubstituted benzimidazoles could possibly generate better antimicrobial activity against *M. tuberculosis*, considering greater equilibrium in the hydrophobic/hydrophilic effect.

### Molecular Docking Study

In order to explore the target of the two series of compounds and provide insights into why they showed disparate antimycobacterial effects, molecular docking was carried out on the FtsZ protein of *M. tuberculosis*. This docking study was based on the binding site previously reported by Li et al. (2017). The protein subunit consists of two globular N- and C-terminal subdomains linked by a central H7 helix having an adjacent T7 loop. FtsZ, a prokaryote tubulin homolog protein, plays a major role in the cytokinesis of mycobacteria. In the presence of guanosine triphosphate (GTP), FtsZ polymerizes to form a dynamic Z-ring inside the cell on the inner membrane and then recruits other cell division proteins (Hong et al., 2013). There is evidence that the disturbance of proper FtsZ assembly would block septum formation and consequently prevent cell division, which should lead to the inhibition of bacterial growth and the promotion of cell death (Slayden et al., 2006; Respicio et al., 2008; Schaffner-Barbero et al., 2012).

**Figure 3:**
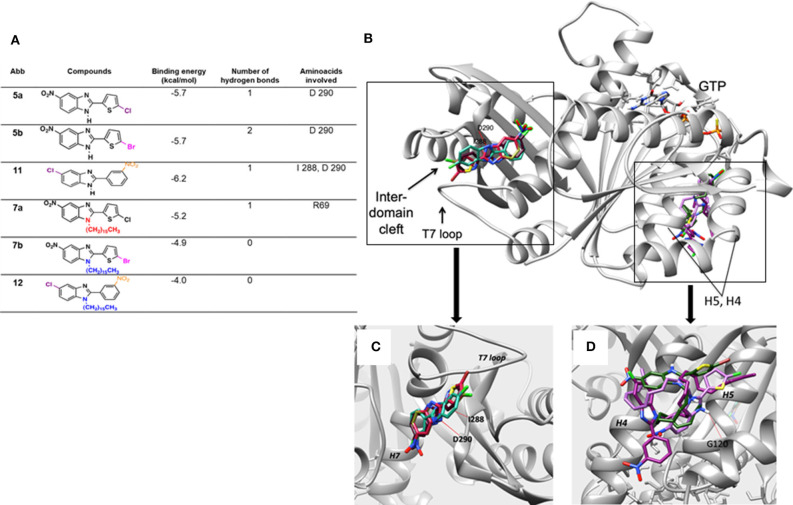
**(A)** Docking score of the binding energy and analysis of the amino acids involved in the interaction. **(B)** Binding pose for the six compounds within the interdomain cleft of FtsZ. Whereas, **5a**, **5b**, and **11** docked into the interdomain cleft site, showing two hydrogen bonds for **11** and one for **5a** and **5b** (red line), **7a**, **7b**, and **12** docked near the GTP binding site. **(C)** Close-up of the binding conformations of compounds **5a**, **5b**, and **11**, which all adopted a similar structure when docking at the interdomain cleft of the FtsZ protein. **(D)** Close-up of the binding domain of compounds **7a**, **7b**, and **12**. They docked between Helix 4 and H5 of the FTsZ protein with a less favorable binding energy than the 2,5-disubstituted compounds. The hydrogen bonds are represented by red lines.

Molecular docking was performed on the six test compounds, which had been found to exhibit different antimycobacterial activity. The compounds were docked to get the best molecular confirmations in the interdomain cleft, which is crucial to GTP-dependent polymerization of the FtsZ protein. The binding energy and H-bonding were taken as two important parameters of the interactions (Figure 3).

Based on the molecular docking study, the three 2,5-disubstituted benzimidazoles, **5a**, **5b**, and **11**, adopt a similar arrangement. The halogens are exposed to FtsZ and the hydrophobic groups interact with the T7 loop of the protein. Two hydrogen bonds were detected for **11** and only one for **5a** and **5b**. Thus, the former showed a better binding energy (−6.2 kcal/mol). The main amino acids in the protein that established hydrogen bonds with the benzimidazoles were I 288 and D 290 (Figure 3). The molecular docking results for **5a**, **5b**, and **11** are consistent with previous reports. The benzimidazole scaffold and cyclohexyl group were observed inside the hydrophobic environment, with the halogen groups exposed to the cleft of FtsZ (Ding et al., 2016).

Contrarily, the three 1,2,5-trisubstituted derivatives containing hydrocarbon chains, **7a**, **7b**, and **12**, do not dock at the same domain nor show strong affinity. Rather, they dock to the N-terminal side of the protein close to the GTP binding domain. Structurally, they interact with the domain formed by helices four and five (H4 and H5). Because of their size, they are not able to position themselves in any cavity of the protein. Both binding affinity and docking results correlated with the antimicrobial effective dose.

## Experimental Protocols

### General Methods

4-Nitro-1,2-phenylenediamine, 4-chloro-2-nitroaniline, 2-thiophenecarboxaldehyde, 3-nitrobenzaldehyde, 2-pyridinecarboxaldehyde, N-bromosuccinimide, and N-chlorosuccinimide (Sigma-Aldrich, St. Louis, MO, USA), were used for syntheses without additional purification. The organic solvents were purified by the standard methods. The progress of each reaction was followed by thin-layer chromatography (TLC) on silica plates (silica gel 60 F_254_, Merck) and with UV light, iodine vapor, or an acidic aqueous solution of KMnO4 for visualization. Column chromatography was performed on silica gel (70–230 mesh, Sigma-Aldrich) with freshly distilled hexane/ethyl acetate as the eluent. Dimethylformamide (DMF) was dried with CaH_2_ at reflux. Acetonitrile was dried with phosphorous pentoxide under constant stirring at rt. Melting points were determined on an electrothermal melting point apparatus. Infrared spectra (FT-IR) were recorded on a double-beam Perkin-Elmer Model 1605 FT-IR spectrometer. NMR spectra were recorded in acetone-d_6_, DMSO-d_6_, or CDCl_3_ solution on Gemini 200, Eclipse 300, or Agilent 500 spectrometers operating at 200, 300, and 500 MHz for ^1^H NMR and 50, 75, and 125 MHz for ^13^C NMR, respectively. Chemical shifts are described in parts per million (ppm), employing Me_4_Si as the internal standard. Coupling constants (*J*) are expressed in Hz. Mass spectra were recorded on a double-beam JEOL JMS AX505HA spectrometer in the electron impact mode and high-resolution mass spectrometry (HR-MS) with electrospray ionization (Bruker Daltonics, Billerica, MA, USA). All experiments were carried out at rt.

### Synthesis of Benzimidazoles

The benzimidazole derivatives were synthesized by two different chemical pathways, one of which was a modification of the method proposed by Abdelhamid et al. (1988), who used phenylenediamine or phenylenediamine with electron-donating substituents. Briefly, 2-thiophenebenzaldoxime (**2**) was synthesized by stirring a mixture of 2-thiophencarboxaldehyde and hydroxylamine hydrochloride in basic medium for 2 h. Subsequently, **2** was treated with NCS or NBS in dry DMF at 15–20°C for 2 h to form **3a** or **3b**.

For the synthesis of the new benzimidazoles, the key intermediate (2-thiophenhydroxymoyl halides **3**) was reacted without purification with 4-nitro-1,2-phenylenediamine (**4**) in dry DMF at 80°C for 6 h to obtain **5a** and **5b**. The novel benzimidazoles **7a**, **7b**, and **12** were synthesized by N-alkylation of **5a**, **5b**, and **11** with hexadecyl methanesulfonate **6** and sodium bicarbonate in dry acetonitrile and heating at reflux for 4 h.

The second method was only for the synthesis of **11** and **12**. 4-Chloro-1,2-phenylenediamine **9** served as a key intermediate, prepared from the reduction of 4-chloro-2-nitroaniline **8** with sodium metabisulfite (Mckinney et al., 1962; Naeimi and Alishahi, 2014). Compound **9** was mixed with 3-nitrobenzaldehyde **10** in dry acetonitrile at rt, using bentonite clay as a heterogeneous catalyst, to give **11** (Cardozo et al., 2015). The latter was N-alkylated with hexadecyl methanesulfonate **6** basic medium and heated at 120°C for 4 h to afford the trisubstituted benzimidazole **12**.

### Alamar Blue Susceptibility Test

The mycobacterial effect of benzimidazole derivatives was evaluated on the *M. tuberculosis* H37Rv strain by performing microcolorimetric assays with Alamar Blue dye, according to Collins and Franzblau (1997). Briefly, the four benzimidazoles at concentrations in the range of 200–3.125 μg/mL were tested in 96-well sterile microplates. Isoniazid was included as the reference drug. Stock solutions of the benzimidazoles were prepared in dimethylsulfoxide at 20 mg/mL in sterile conditions and stored at −70°C to await further use. Standard solutions were obtained by consecutive twofold dilutions of the stock solutions in 7H9 Middlebrook medium until reaching 800 μg/mL, followed by the addition of 100 μL of Middlebrook 7H9 broth, 100 μL of the benzimidazole solution and 100 μL of mycobacterial suspension to each well. A 1:10 dilution was made for the control from the bacterial suspension, representing the growth of 10% of the bacterial population assayed. After incubating the plates at 37°C for 5 days, 20 μL of Alamar Blue solution (AbD Serotec) and 12 μL of sterile 10% Tween 80 were added. The plates were incubated at 37°C for another 24 h before the determination was made, considering wells with a pink color darker than that of the 10% control well to be positive for growth. The minimal inhibitory concentration (MIC) was defined as the lowest at which benzimidazole impeded development of the pink color.

### Intracellular Activity of the Benzimidazoles

In order to examine the intracellular antimycobacterial activity, monolayers of mouse macrophages (J774A.1) were infected and then treated in independent assays with each benzimidazole or isoniazid. Macrophage monolayers were prepared by utilizing 2.5 × 10^5^ cells/mL in RPMI-1640 medium (Sigma-Aldrich) supplemented with 10% fetal bovine serum (HyClone®, Thermo SCIENTIFIC) and placed in 24-well plates. The plates were incubated at 37°C in a humidified 5% CO_2_ atmosphere until the monolayers reached 90% cell confluency. Subsequently, the monolayers were washed with Hanks' balanced salt solution (HBSS) to remove the non-adhered cells. To infect the cells, the bacterial suspensions were adjusted to a multiplicity of infection (MOI) ratio of 1:10, and 1 mL of the suspension was added to each well. After infected monolayers were incubated at 37°C under 5% CO_2_ for 2 h, monolayers were washed three times with HBSS to eliminate the bacteria not internalized, followed by the addition of a 1-mL solution of one of the compounds or isoniazid diluted in RPMI-1640 medium. Whereas, all test compounds were evaluated at MIC, +MIC, and –MIC, isoniazid was assayed at 0.5, 0.25, and 0.125 μg/mL. Untreated infected cells were included as controls. The cells were incubated at 37°C under 5% CO_2_, and the kinetics of the activity was monitored at 24, 48, 72, and 96 h. At each time point, cells were lysed by the adding of 500 μL of 0.25% SDS and neutralized with 500 μL of 5% bovine serum albumin (BSA) solution. Serial dilutions of the cell lysates were plated on 7H11 Middlebrook agar supplemented with 10% OADC (oleic acid, albumin, dextrose and catalase) and incubated at 37°C for 3 weeks to determine the number of colony-forming units (CFUs).

### Effect of Benzimidazoles on the Viability of Macrophages

In order to assess the possible toxicity of benzimidazole derivatives to macrophages, a viability assay was performed. Macrophage monolayers were prepared as aforementioned (in subsection intracellular activity of the benzimidazoles) up to washing with HBSS to remove the non-adhered cells. Subsequently, 1 mL of the 50 μg/mL solution of one of the benzimidazoles was added. Some wells were maintained without treatment (viability control), and to others were added 40 μg/mL ursolic acid (death control). The cells were incubated for 24 h at 37°C under 5% CO_2_. Upon completion of this time, 100 μL of Alamar Blue solution was added, the monolayers were incubated again until the viability control wells turned pink. The relative fluorescence units were quantified in a Fluoroskan Ascent FL Microplate fluorometer, and the RFUs of the control wells without treatment were taken as 100% viability.

### Molecular Docking

The molecular docking simulations were carried out on Autodock Vina 4.2.6, which has been calibrated and tested with molecular dynamics by using a set of small-protein ligands of medicinal interest (Perryman et al., 2015; Forli et al., 2016). This software maximizes the accuracy of predicting the binding of small molecules to proteins, assessing the veracity of the predictions by comparing them to the experimental structure. Evaluation of the difference between the experimental and predicted structures considers symmetry, partial symmetry, and near-symmetry (Trott and Olson, 2010).

The X-ray structure of *M. tuberculosis* eukaryotic tubulin homolog protein FtsZ (2.08Å resolution, PDBID 1RLU) (Leung et al., 2004) was obtained from the Protein Data Bank database (http://www.pdb.org). The three-dimensional structures of the ligands were built and their energy minimized with MarvinSketch 18.24.0 ChemAxon software (http://www.chemaxon.com). The ligands and protein were then prepared for molecular docking by adding hydrogens and Gasteiger charges with Autodock tools 1.5.6 (Sanner, 1999). A grid box of 30 Å on each side was placed into the dimer interdomain with grid points separated by 1.0 Å centered at the middle of the protein at *x* = 6.0, *y* = 29, and *z* = 27. The binding energies of the docked complexes were provided by the output Autodock Vina file and the ligand-protein binding interactions at the binding sites were analyzed on UCFS Chimera software (Pettersen et al., 2004).

## Conclusion

The new 2,5-disubstituted benzimidazole derivatives were successfully synthesized by simple, efficient and inexpensive procedures. The synthetic procedure for the 1,2,5-trisubstituted compounds was longer and led to low yields. The structures of all test compounds were fully characterized by FT-IR, NMR, and HR-MS. According to the *in vitro* antimycobacterial assays, the 2,5-disubstituted benzimidazoles exhibited excellent antimycobacterial activity. After adding the hydrocarbon chain in the 1-position of the benzimidazole ring to form the 1,2,5-trisubstituted benzimidazoles, contrarily, a poor antimycobacterial effect was found. The docking analysis of the possible molecular target and mechanism of action of the test compounds suggests that the 2,5-disubstituted benzimidazoles could effectively interact with a crucial domain for the polymerization of the FtsZ protein. On the other hand, the larger molecular size of the 1,2,5-trisubstituted benzimidazoles apparently caused steric restriction, impeding their approach to the putative binding site of the FtsZ protein to block septum formation and cell division. Rather, the 1,2,5-trisubstituted derivatives docked to N-terminal side of the protein, closer to the GTP binding domain. Overall, these results point to the promising antimycobacterial activity of 2,5-disubstituted benzimidazoles. Further *in vivo* studies are needed to determine their potential for treating tuberculosis.

## Author Contributions

RJ-J and BG-P conceptualized and designed the study. RJ-J, NJ-R, GC-R, IU-G, and GM-M carried out the chemical synthesis and characterization of the new compounds. WC-C, RR-N, and NC-J performed the biological activity assays. CA-A was responsible for the docking study. RJ-J, BG-P, NC-J, and CA-A analyzed the results of the investigation. RJ-J, NC-J, CA-A, and BG-P took charge of writing the original draft, which was analyzed for modification by all authors. BG-P edited the following versions of the manuscript, administered the project, and acquired funding.

## Conflict of Interest

The authors declare that the research was conducted in the absence of any commercial or financial relationships that could be construed as a potential conflict of interest.
