# Supplementary material for: Synthesis and Antimycobacterial Activity of 2,5-Disubstituted and 1,2,5-Trisubstituted Benzimidazoles
Source: Front Chem. 2020 Jun 19;8:433. doi: 10.3389/fchem.2020.00433 (PMC7325987; doi:10.3389/fchem.2020.00433)
Supplement: Supplementary file 1 [file Data_Sheet_1.docx]

Supplementary Material

# Supplementary Data

*Thiophene-2-carbaldehyde oxime* ***2***

To prepare a solution of thiophene-2-carboxaldehyde **1** (1.0 equiv, 4.46 mmol); (MeOH/H_2_O/ice-water, 1:1:2), NH_2_OH.HCl (1.2 equiv, 5.35 mmol) and AcONa (2.5 equiv, 11.5 mmol) were mixed (Alam et al., 2011). The reaction was maintained in continue stirring. The progress of the reaction was followed by TLC ethyl acetate/hexane, 1:3 as eluent. After completion of the reaction, the organic solvent was removed under vacuum. The water face was extracted with ethyl acetate (3x40 mL). The extracts were gathered together, dried and evaporated to dryness. Compound **2** was obtained as white solid, (0.526 g, 93 %); mp 137-138 °C, (Previous reported, 136 °C) (Iwakura et al., 1968). Rf 0.29 ethyl actetate/hexane, 1:3, UV. IR (KBr): 3025 (OH), 1632 (C=NOH). ^1^H NMR (500 MHz, CDCl_3_): δ = 7.11 (dd, 1H, *J* = 6 y 8 Hz, H_4_); 7.42 (dd, 1H, *J* = 2 y 6 Hz, H_5_); 7.58 (dd, 1H, *J* = 1.5 y 8 Hz, H_3_); 7.76 (s, 1H, H-C=NOH); 9.51 (br, 1H, H-O). ^13^C NMR (125 MHz, CDCl_3_): δ = 131.9 y 131.7, 131.2, 129.5, 127.8, 126.2.

*5-Chlorothiophene-2-carbohydroximoyl chloride* ***3a***

The synthesis of compound **3a** was made combining the procedures of Iwakura et al., (1968) and Kanemasa et al., (2000). To prepare a solution of thiophene-2-carbaldehyde oxime **2**, 3.11 g (1.0 equiv, 24.5 mmol) in dry dimethylformamide (DMF, 4 mL) (CaH_2_) was cooled in water-ice bath to 15 °C. NCS 9.83 g (3.0 equiv, 73.5 mmol) was added in portion wise during 1 h. The progress of the reaction was followed by TLC ethyl acetate/hexane (1:4). The reaction ended after 4 h. Then, reaction mixture was poured onto water-ice mixture 1:1 (50 mL) and extracted with dichloromethane (3x30 mL). Organic extracted were dried (Na_2_SO_4_) and the solvent evaporated to vacuum. The compound **3a** was obtained as yellow solid (4.56 g, 95 %); mp 129.5 °C, (Previous reported 127-128 °C) (Iwakura et al., 1968). Rf 0.45 ethyl acetate/hexane 1:4, UV.

*5-Bromothiophene-2-carbohydroxamoyl bromide* ***3b***

Compound **3b** was obtained using: thiophene-2-carbaldehyde oxime **2** 3.11g (1.0 equiv, 24.5 mmol), dry DMF (4 mL), NBS 13.08 g (3.0 equiv, 73.5 mmol). It was obtained as oily brown solid (0.417 g, (93 %). Rf 0.43 ethyl acetate/hexano 1:4, UV.

*2-(5’-chlorothien-2’-yl)-5(6)-nitrobenzimidazole* ***5a***

The benzimidazoles **5a** and **5b** were made modifying the Abdelhamid's method (Abdelhamid et al., 1988) as follow. The 2-chlorothiophene-2-carbohydroximoyl chloride **3a** 0.360 g (1.0 equiv., 1.8367 mmol) and 4-nitro-1,2-phenylenediamine 0.225 g (0.8 equiv., 1.4706 mmol) were dissolved in dry DMF (10 mL) and were heated at 80 °C for 6 h. The reaction progress was followed by TLC ethyl acetate/hexane 2:3, UV. After, the solvent was evaporated at reduced pressure. The reaction crude obtained was dissolved in acetone, absorbed in silica gel and separated by silica gel (70-230 meshes) column chromatography, using mixtures in gradient of ethyl acetate/hexane as mobile phase. The compound **5a** was obtained as amorphous beige solid in 60 % (0.2462 g) yield, mp 262-263 °C, Rf 0.29 ethyl acetate/hexane 2:3, UV

IR (KBr) ʋ 3257 (N-H), 3098 (H-C=), 1578 (N=C), 1506 (NO_2_) cm^-1^

^1^H NMR (300 MHz, (D_3_C)_2_CO-d_6_), δ: 12.56 (s, 1H, H-N); 8.44 (d, 1H, *J*=2.4 Hz, H_4_); 8.17, 8.14 (dd, ^1^H, *J*=8.7, 2.4 Hz, H_6_); 7.76 (d, 1H, *J*=3.9 Hz, H_3´_); 7.72 (d, 1H, *J*=10Hz, H_7_); 7.19 (d, ^1^H, *J*=3.9Hz, H_4´_).

^13^C NMR (75 MHz, (D_3_C)_2_CO-d_6_)), δ: 150.5; 144.5; 132.6; 128.9; 128.2; 119.7; 119.5; 118.8; 115.8; 112.1, 108.5 ppm.

MS (EI, 70 eV), m/z (%): 279 [M+ (100)]; 281 [M^+^.+2, (35)]; 249 [M^+^.-NO, (28)]; 233 [M^+^.-NO_2_, (32)].

HR-MS (m/z) experimental molecular weight (M+1) 279.9942 g/mol. Calculated molecular weight 279.9947 g/mol.

*2-(5’-bromothien-2’-yl)-5(6)-nitrobenzimidazole* ***5b***

Benzimidazole **5b** was synthesized using the following quantities. 2-Bromothiophene-2-carbohydroximoyl bromide **3b** 0.5 g (1.0 equiv., 1.7544 mmol), 4-nitro-1,2-phenylenediamine 0.2147 g (0.8 equiv., 1.4035 mmol) and dry DMF (10 mL). The compound **5b** was obtained as amorphous brown solid in 98 % (0.4455 g) yield, mp 254-255 °C, Rf 0.2932 ethyl acetate/hexane 1:2, UV.

IR (KBr) ʋ 3385 (N-H), 1625 (N=C), 1573, 1337 (NO_2_) cm^-1^

^1^H NMR (200 MHz, DMSO-d_6_), δ: 8.38 (s, 1H, H4), 8.09 (d, 1H, *J*=7 Hz, H_6_), 7.70 (s, 2H, H_7_, H_3’_), 7.38 (s, 1H, H_4’_).

^13^C NMR (50 MHz, DMSO-d_6_), δ: 150.1, 142.8, 134.0, 132.0, 128.9, 118.2 y 116.1, 114.4, 111.8.

EM (EI, 70 eV), m/z (%): 323 [M+, (95)], 325 [M^+^.+2, (100)], 277 [M^+^.-NO_2_, (24)], 279 (M^+^.+2-NO_2_, (22)].

HR-MS (m/z) experimental molecular weight (M+1) 323.9291 g/mol. Calculated molecular weight 323.9442 g/mol.

*4-Chloro-1,2-phenylenediamine* ***7***

4-chloro-2-nitroaniline **6** 0.5 g (1.0 equiv., 2.9 mmol) and sodium metabisulfite 2.02 g (4 equiv., 11.609 mmol) were suspended in water (5 mL) and were refluxed for 2 h. The reaction progress was followed by TLC ethyl acetate/hexane (1:3), UV. At the end, was added water (40 mL) and extracted with ethyl acetate (3x30 mL). The organic phase was dried with sodium sulphate, filtered, and the organic solvent evaporated at the vacuum. The 4-chloro-1,2-phenylenediamine **7** was obtained as an amorphous beige solid in quantitative yield and was not necessary to purified it. Physical and spectroscopic properties were similar to previously reported (Cantillo et al., 2013).

*2-(3’-nitrophenyl-1-yl)-5(6)-chlorobenzimidazol* ***11***

4-Chloro-1,2-*o*phenylenediamine **7** 0.4130 g (2.9 mmol, 0.8 equiv.), 3-nitrobenzaldehyde 0.5470 g (3.6 mmol, 1.0 equiv.) and bentonite clay 0.5470 g were suspended in dry acetonitrile (15 mL) and stirred at room temperature for 24 h. The reaction progress was followed by TLC, ethyl acetate/hexane (1:3), UV. After the start materials were consumed, the organic solvent was evaporated at vacuum. The residue obtained was dissolved in acetone and filtered through celite. Then, the solvent was evaporated at reduced pressure and the crude product was purified by silica gel (70-230 mesh) column chromatography, using ethyl acetate/hexane (5:95 and 40:60) as mobile phase. The benzimidazol **9a** was obtained as amorphous beige solid in 64 % yield (0.419 g), mp 243-244°C, Rf 0.45 ethyl acetate/hexane (1:3) UV. Spectroscopic properties were similar to previously reported (Keurulainen et al., 2010).

IR (KBr), ʋ: 3322 (N-H), 1513 (NO_2_) cm^-1^

^1^H NMR, δ: 13.4 (sa, 1H, NH), 8.96 (t, 1H, *J*=1.2 Hz, H-2’), 8.5 (dt, 1H, *J*=7.8, 1.2 Hz, H-6’), 8.3 (dt, 1H, J=7.8, 1.2 Hz, H-4’), 7.8 (t, 1H, J=7.8 Hz, H-5’), 7.6 (t, 2H, J=8.4 Hz, H-4, H-7), 7.2 (dd, 1H, *J*=8.4, 2.1 Hz, H-6).

RMN ^13^C, δ: 151.1, 149.0, 133.2, 131.9, 131.4, 125.2, 123.6, 121.6.

EM (m/z, %): 275 (M^+^+2, 33), 273 (M^+^, 100), 243 (M^+^-NO, 4), 227 (M^+^-NO_2_, 45), 192 (M^+^-NO_2_ y Cl, 30).

*1-hexadecyl-2-(5’-chlorothien-2’-yl)-5(6)-nitrobenzimidazole* ***7a***

2-(5´-chlorothien-2-yl)-5(6)-nitrobenzimidazole **5a** 1.86 g (6.6 mmol. 1.0 equiv.) were dissolved in DMF (6 mL), sodium bicarbonate 1.68 g (20.0 mmol, 3.0 equiv.) were added in a single portion. The solution was stirred for 4.0 h to room temperature. Then, hexadecyl methanesulfonate 2.56 g (8.0 mmol, 1.2 equiv.) and the mixture reaction were heated at 120 °C. The reaction progress was followed by TLC, ethyl acetate/hexane 1:4, UV. When the start materials not changed, the reaction mixture was poured in water (100 mL). The solid crude product was filtered off and then extracted by stirring with excess hexane (80 mL) during 20 min a room temperature. The mixture obtained was filtered and washed with hexane (3x10 mL), for to give the benzimidazole **5a** (60% recovery). The organic phase was evaporated and the solid crude product was purified by silica gel column chromatography. The trisubstituted benzimidazole **7a** was obtained as amorphous beige solid 35% yield (0.704 g); mp 93-94 °C.

IR (KBr) ʋ: 2916, 2848 (C-H), 1520 (C=N), 1472 y 1462 (NO2) cm^-1^

^1^H NMR (CDCl_3_, 500 MHz) δ: 8.31 (bs, 1H, H_4_); 8.21 (bdd, 1H, *J*=10 Hz, H_5_); 7.79 (d, 1H, *J*=10 Hz, H_7_); 7.41 (d, 1H, *J*=5 Hz, H_3’_); 7.05 (d, 1H, *J*=5 Hz, H_4’_); 3.62 (t, 2H, *J*=5.0 Hz, H_2_C-N); 1.33, 1.24 (bs, 24H, (CH_2_)_14_), 0.86 (t, 3H, *J*=5.0 Hz, CH_3_).

*1-hexadecyl-2-(5’-bromothien-2’-yl)-5(6)-nitrobenzimidazole 7b*

Benzimidazole **7b** was synthesized using the following quantities. 2-(5’-bromothien-2-yl)-5-nitrobenzimidazole **5b** 1.0 g (3.08 mmol, 1.0 equiv.), dimethylformamide (6.0 mL), sodium bicarbonate 0.5185 g (6.17 mmol, 2.0 equiv.), hexadecyl methanesulfonate 1.1851 g (3.70 mmol, 1.2 equiv.). The benzimidazole **5b** unreacted 60% yield (0.6 g). The benzimidazole **7b** was obtained as amorphous beige solid 30% yield (202.9 mg); mp 92-93 °C.

IR (ATR) ʋ: 2917, 2850 (C-H), 1614 (C=N), 1519 y 1331 (NO_2_) cm^-1^

^1^H NMR (CDCl_3_, 500 MHz) δ: 8.39 (bs, 1H, H_4_); 8.28 (td, 2H, *J*=5, 10 Hz, H_5_); 7.88 (d, 1H, *J*=10 Hz, H_7_); 7.73 (d, 1H, *J*=5 Hz, H_3’_); 7.69 (d, 1H, *J*=5 Hz, H_4’_); 3.98 (t, 2H, *J*=5 Hz, H_2_C-N); 1.41, 1.31 (bs, 24H, (CH_2_)_14_), 0.93 (t, 3H, *J*=5 Hz, CH_3_).

*1-hexadecyl-2-(3’-nitrophenyl-1’-yl)-5(6)-chlorobenzimidazole* ***12***

The compound **12** was synthesized following the same procedure to synthesis of benzimidazole **7a**. The synthesis included the following quantities: 2-(3’-nitrophenyl-2’-yl)-5-chlorobenzimidazole **11** 0.11 g (0.4029 mmol, 1.0 equiv.), DMF (6.0 mL), sodium bicarbonate 0.1015 g (1.2088 mmol, 3.0 equiv.), hexadecyl methanesulfonate 0.1547 g (0.4835 mmol, 1.2 equiv.). The trisubstituted benzimidazole **12** was obtained as viscous cherry solid 18% yield (36 mg); mp 95-96 °C.

^1^H NMR (CDCl_3_, 500 MHz) δ: 8.59 (bs, 1H, H_2´_); 8.39 (bdd, 1H, *J*=5 Hz, H_4´_); 8.13 (bd, 1H, *J*=5 Hz, H_6´_); 7.82 (bd, 1H, *J*=5 Hz, H_4_); 7.75 (t, 1H, *J*=5 Hz, H_5’_); 7.35 (t, 2H, *J*=10 Hz, H_5´_); 7.34 (bd, 1H, *J*=10 Hz, H_7_); 3.64 (t, 2H, *J*=5 Hz, H_2_CN); 1.55 (q, 2H, *J*=5 Hz, H_2_CC_2_N); 1.25 (bs, 28H, (CH_2_)_14_), 0.88 (t, 3H, *J*=5 Hz, CH_3_).

**References**

Abdelhamid, A.O., Párkányi, C., Rashid, S. M. K., Lloyd, W.D. (1988). Synthesis of fused ring heterocycles from aromatic amines with hydroximoyl chlorides. J. Heterocycl. Chem. 25, 403-405. doi.org/10.1002/jhet.5570250210.

Alam, A., Pal, C., Goyal, M., Kundu, M.K., Kumar, R., Iqbal, M.S., Dey, S., Bindu, S., Sarkar, S., Pal, U., Maiti, N.C., Adhikari, S., Bandyopadhyay, U. (2011). Synthesis and bio-evaluation of human macrophage migration inhibitory factor inhibitor to develop anti-inflammatory agent. Bioorg. Med. Chem. 19, 7365-73. doi: 10.1016/j.bmc.2011.10.056.

Cantillo, D., Moghaddam, M. M., Kappe, C. O. (2013). Hydrazine-mediated reduction of nitro and azide functionalities catalized by highly active and reusable magnetic oxide nanocrystals. J. Org. Chem. 78, 4530-4542. doi.org/10.1021/jo400556g.

Iwakura, Y., Uno, K., Shiraishi, S., Hongu, T. (1968). 1,3-Dipolar cycloaddition reaction of thiophenecarbonitrile N-oxides. Bull. Chem. Soc. Jap. 41, 2954-2959. doi.org/10.1246/bcsj.41.2954

Kanemasa, S., Matsuda, H., Kamimura, A., Kakinami, T. (2000). Synthesis of hydroximoyl chlorides from aldoximes and benzyltrimethylammonium tetrachloroiodate (BTMA ICl4). Tetrahedron. 56, 1057-1064. doi.org/10.1016/S0040-4020(99)01047-9

Keurulainen, L., Salin, O., Siiskonen, A., Kern, J. M., Alvesalo, J., Kiuru, P., Maass, M., Yli-Kauhaluoma, J., Vourela, P. (2010). Design and synthesis of 2-arylbenzimidazoles and evaluation of their inhibitory effect against Chlamydia pneumonia. J. Med. Chem. 53, 7664-7674. doi: 10.1021/jm1008083.

# Supplementary Figures and Tables

## Supplementary Figures


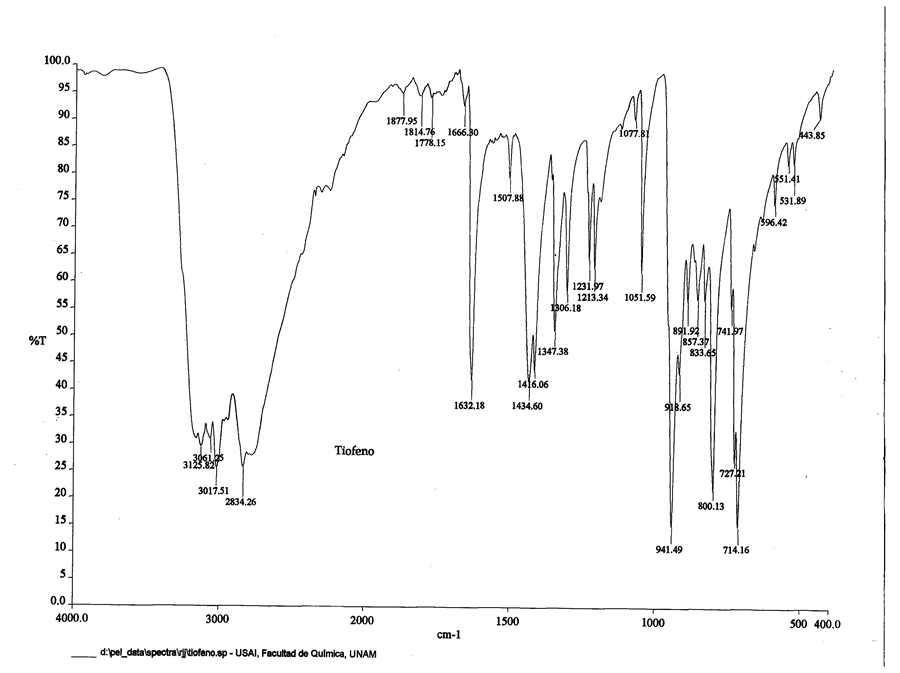


**Supplementary Figure 1.** Infrared spectrum of 2-Thiophenecarboxaldehyde oxime **2**


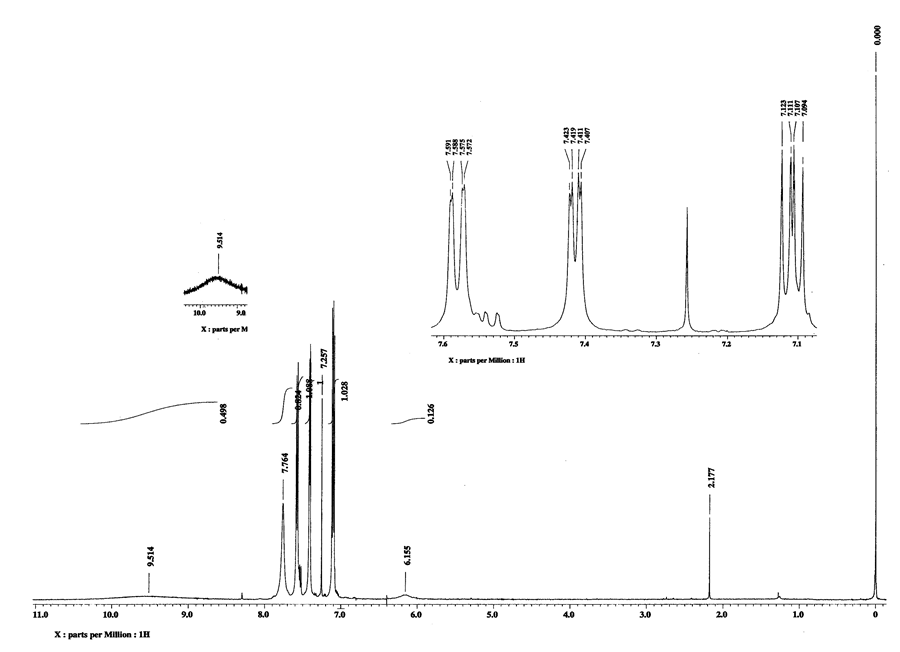


**Supplementary Figure 2.** ^1^H NMR spectrum of 2-Thiophenecarboxaldehyde oxime **2**


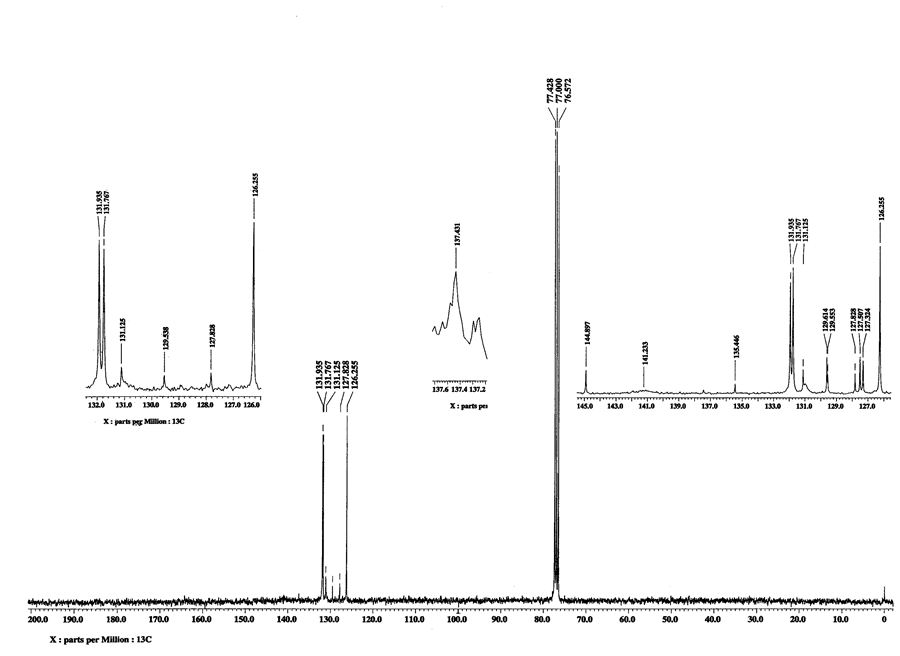


**Supplementary Figure 3.** ^13^C NMR spectrum of 2-Thiophenecarboxaldehyde oxime **2**


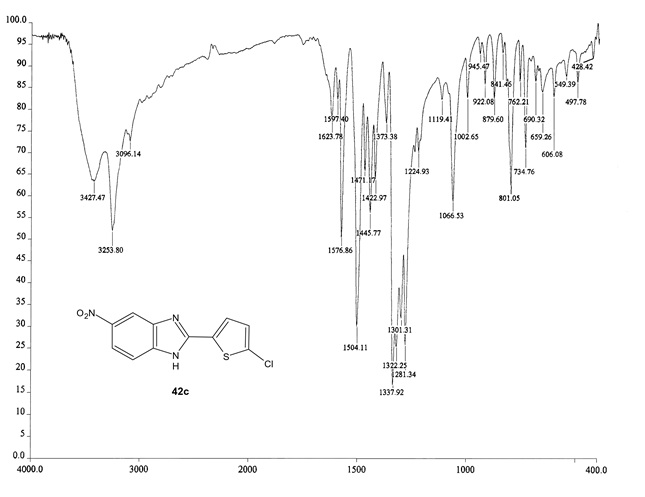


**Supplementary Figure 4.** Infrared spectrum of 2-(5’-chlorothiophene-2’-yl)-5-nitrobenzimidazole **5a**


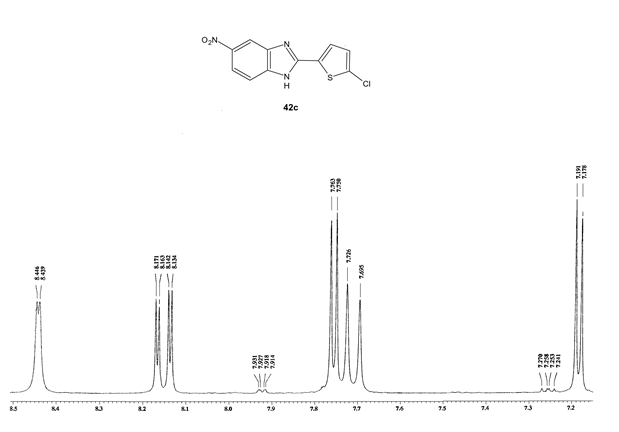


**Supplementary Figure 5.** ^1^H NMR spectrum of 2-(5’-chlorothiophene-2’-yl)-5-nitrobenzimidazole **5a**

**
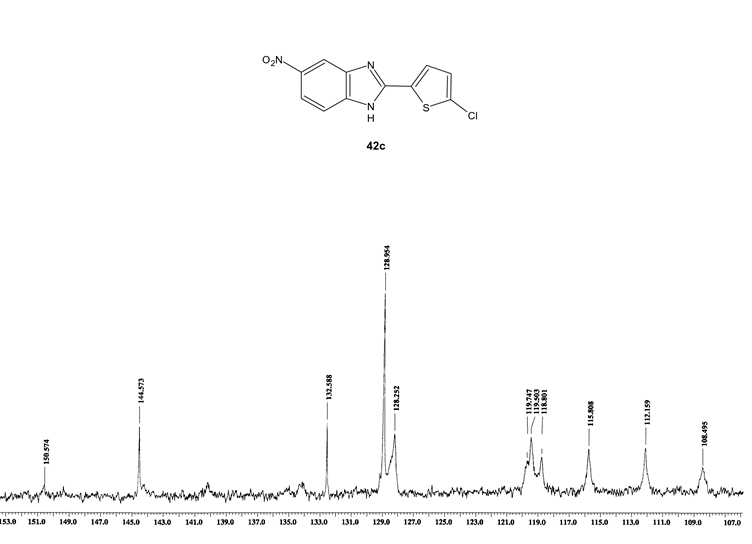
**

**Supplementary Figure 6.** ^13^C NMR spectrum of 2-(5’-chlorothiophene-2’-yl)-5-nitrobenzimidazole **5a**


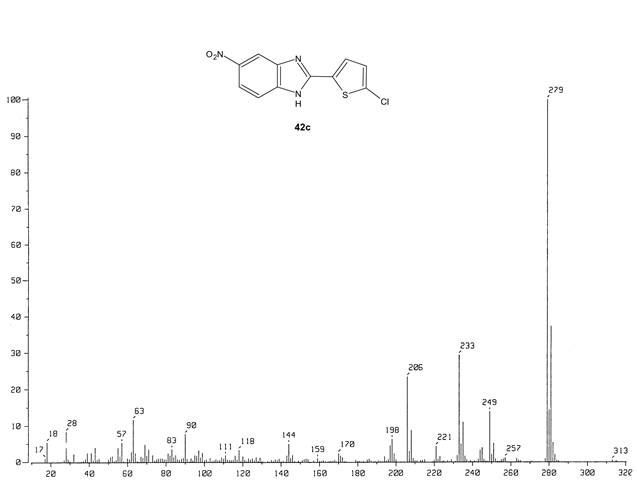


**Supplementary Figure 7.** LR-MS spectrum of 2-(5’-chlorothiophene-2’-yl)-5-nitrobenzimidazole **5a**


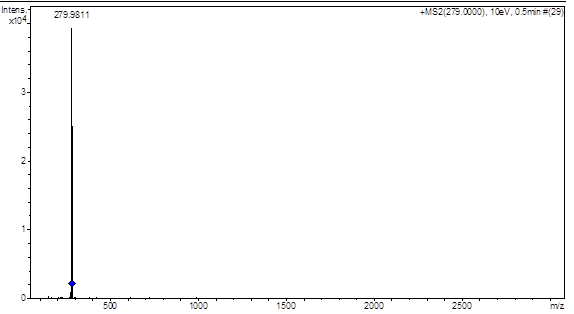


**Supplementary Figure 8.** HR-MS spectrum of 2-(5’-chlorothiophene-2’-yl)-5-nitrobenzimidazole 5a


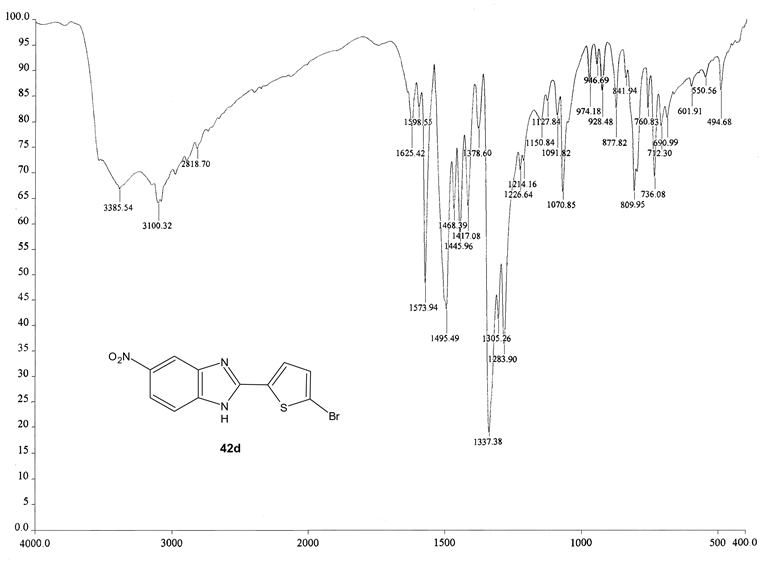


**Supplementary Figure 9.** Infrared Spectrum of 2-(5’-bromothiophene-2‘-yl)-5-nitrobenzimidazole **5b**

**
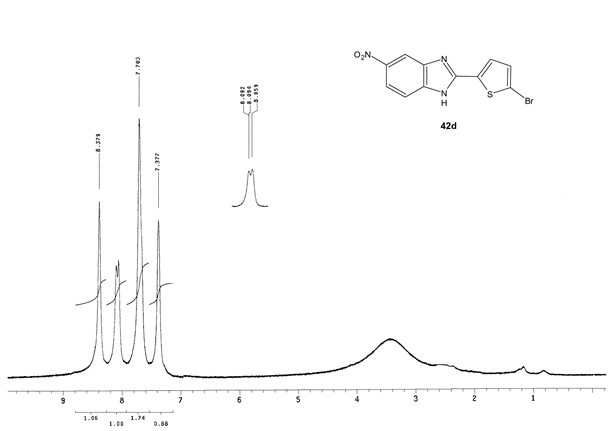
**

**Supplementary Figure 10.** ^1^H NMR Spectrum of 2-(5’-bromothiophene-2‘-yl)-5-nitrobenzimidazole **5b**

**
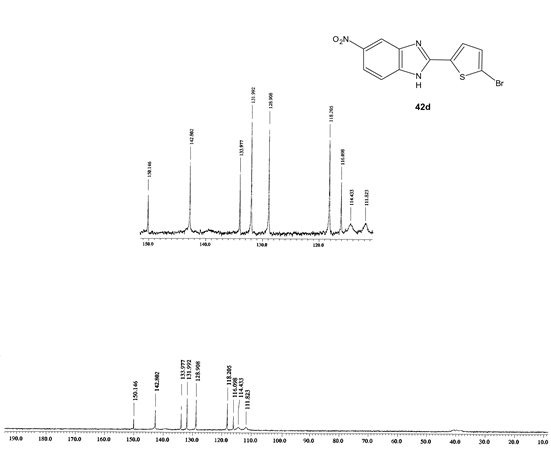
**

**Supplementary Figure 11.** ^13^C NMR spectrum of 2-(5’-bromothiophene-2‘-yl)-5-nitrobenzimidazole **5b**


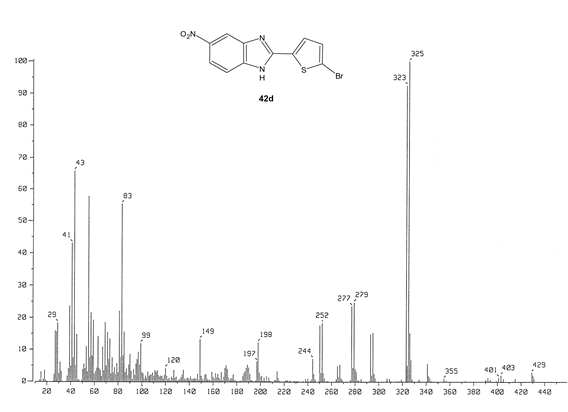


**Supplementary Figure 12.** LR-MS Spectrum of 2-(5’-bromothiophene-2‘-yl)-5-nitrobenzimidazole **5b**


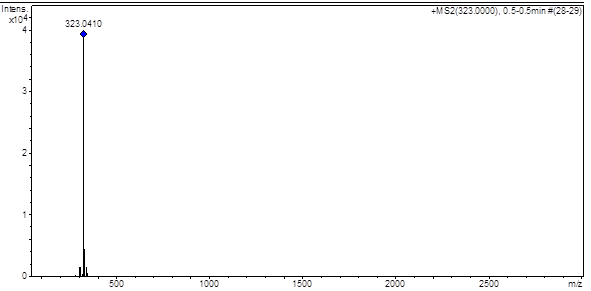


**Supplementary Figure 13.** HR-MS Spectrum of 2-(5’-bromothiophene-2‘-yl)-5-nitrobenzimidazole **5b**


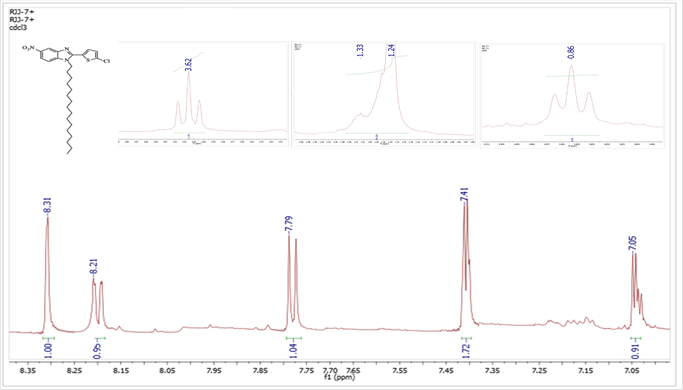


**Supplementary Figure 14.** ^1^H NMR Spectrum of **7a**


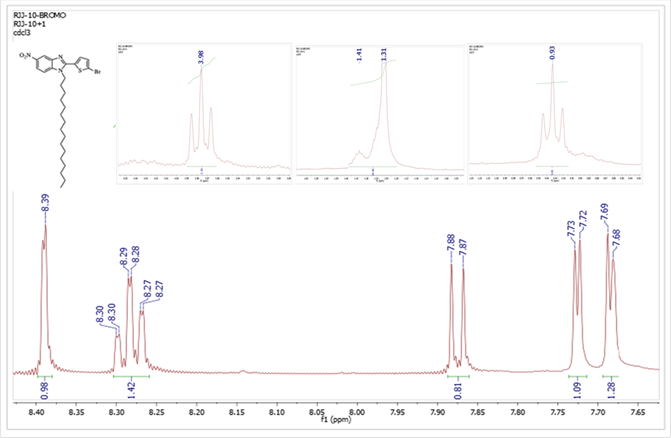


**Supplementary Figure 15.** ^1^H NMR Spectrum of **7b**

**
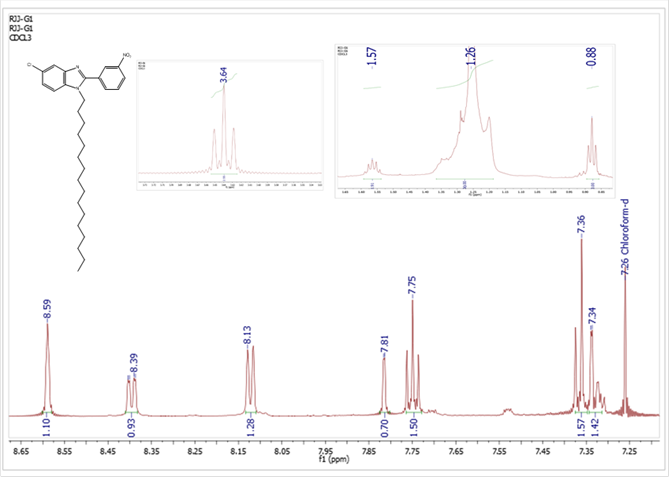
**

**Supplementary Figure 16.** ^1^H NMR Spectrum of **12**
